# Tuberculous Arthritis of the Ankle Joint Mimicking Pigmented Villonodular Synovitis

**DOI:** 10.7759/cureus.13962

**Published:** 2021-03-18

**Authors:** Tareq A Alrefeidi, Ali M Alahmari

**Affiliations:** 1 Orthopedic Surgery, Armed Forces Hospital Southern Region, Khamis Mushayt, SAU; 2 Orthopedic Surgery, King Abdullah Medical City, Mecca, SAU

**Keywords:** ankle, tuberculosis, extrapulmonary tuberculosis, case report, diagnosis

## Abstract

Osteoarticular tuberculosis (TB) of the ankle joint is rare, and there is a delay in diagnosis in most cases. All patients with the disease complained of swelling and pain in the affected ankle. Ankle TB is a disorder that can be simply misdiagnosed. The delay in diagnosis may range from months up to years. All patients in this series had already been diagnosed with ankle TB by the time they visited our hospital. The current case was reported in a middle-aged male nurse with ankle pain, ulcer, and limited range of movement. The classical clinical manifestations of TB were absent on free chest radiography. Ulcer biopsy showed TB infection with no lymph nodes. Nonspecific inflammation was initially treated, but, subsequently, anti-TB treatment was useful.

## Introduction

Tuberculosis (TB) is an infectious disease mostly caused by the bacteria Mycobacterium tuberculosis [1]. TB is mainly pulmonary disease affecting the lungs, but it can also affect other parts of the body [2,3]. Most TB cases may be asymptomatic, in which case it is known as latent TB [4].

The incidence of extrapulmonary TB has also increased. Roughly 10% of extrapulmonary TB cases involve joints and bones, constituting 1-3% of all TB cases. Latent joint and bone TB affects approximately 19-38 million individuals globally [5,6]. TB arthritis is most commonly featured as monoarthritis of the weight-bearing joints, including the hip or knee. Polyarticular effects are reported and may cause misdiagnosis of inflammatory arthritis [7].

## Case presentation

A male patient aged 39 years who works as nurse, smokes, and has hypertension was referred from a general hospital to the outpatient department of our specialized hospital in Mecca, Saudi Arabia. The patient complained of insidious onset pain and swelling in the left ankle joint for the past seven months, which progressed to an open ulcer at the lateral side of the left ankle joint. There was no specific history of trauma, weight loss, night sweats, or anorexia. The patient was initially treated with nonsteroidal anti-inflammatory drugs, with minimal improvement of the symptoms for some time at the referring hospital.

The initial examination revealed diffuse swelling and tenderness around the left ankle joint with mild hotness and no fever or redness. The patient could not tolerate weight-bearing activity and had a significantly decreased range of motion. The patient had calf muscle atrophy.

**Figure 1:**
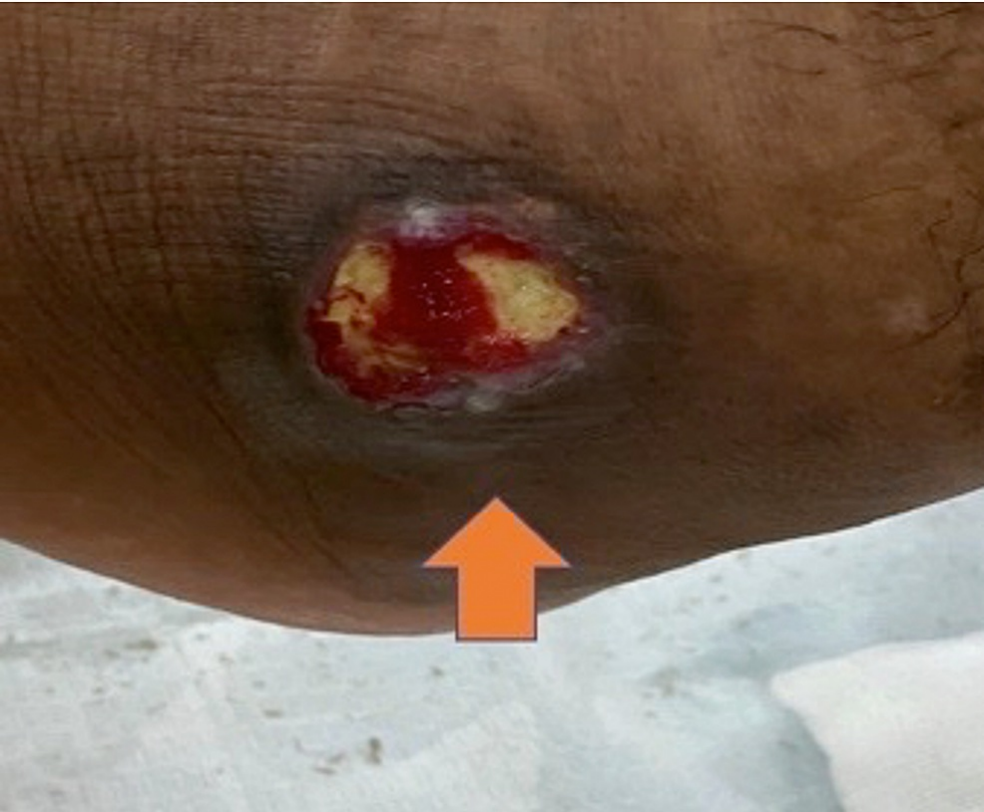
Lateral side image of the left ankle showing ulcer

There was a 3 × 3 cm circular ulcer with sharp edges at the lateral side of the left ankle with no active discharge (Figure 1).

Laboratory investigations showed a high alkaline phosphate level (157.0 U/L), high WBC count (11.92 × 10^9^/L; mainly lymphocytes), high erythrocyte sedimentation rate (21 mm/h), and C-reactive protein level of 0.91 mg/dL.

**Figure 2:**
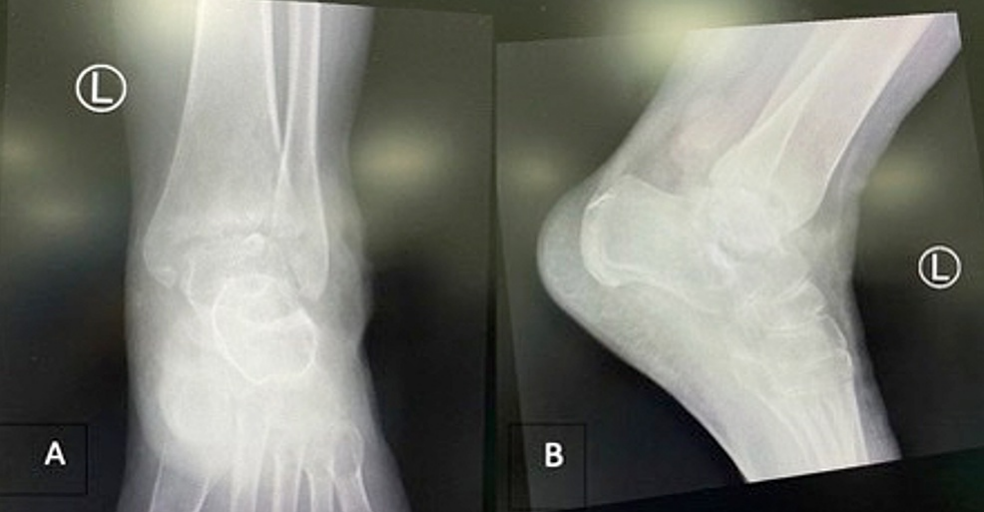
(A) Anteroposterior and (B) lateral plain radiographs showing mild arthritic changes and soft tissue swelling

Anteroposterior and lateral plain radiographs of the left ankle showed mild arthritic change of the tibiotalar joint and soft tissue swelling (Figure 2).

**Figure 3:**
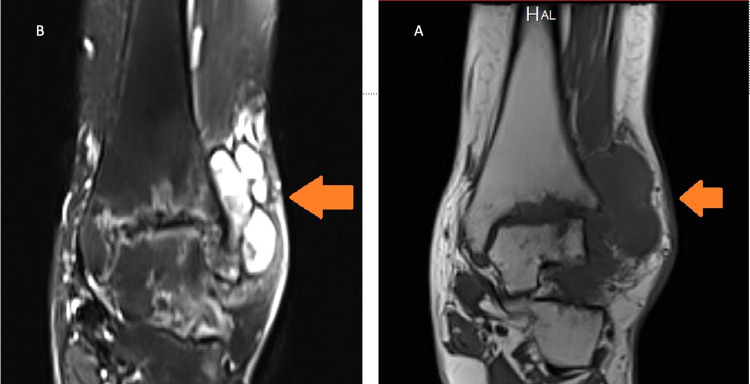
(A) T1- and (B) T2-weighted coronal view MRI of the left ankle showing multinodular mass and low signal intensities indicating that hemosiderin deposits were diagnostic of diffuse pigmented villonodular synovitis

Magnetic resonance imaging (MRI) revealed extensive low T1/T2 signal infiltrative synovial lesions filling most of the tibiotalar/subtalar joints spaces, which were associated with sizable effusion-filled anterolateral/posterolateral joint recess. There were subsequent diffuse, uniform secondary advanced tibiotalar joint osteoarthritic changes, which was associated with multiple variable-sized osseous erosion, which was highly concerning for underlying diffuse pigmented villonodular synovitis (PVNS) (Figure 3).

There were no clinical or radiological signs of TB, and chest radiographs were clear.

Then, we performed open wound biopsy from the ulcer with sterile condition, which revealed TB. Also, no PVNS was detected.

The patient was referred to an infectious disease physician, and anti-TB medication was prescribed (ethambutol 1,200 mg orally once daily, rifampicin 600 mg orally once daily, isoniazid 300 mg orally daily, and pyrazinamide 1,000 mg orally once daily) for nine months, and follow-up showed improvement in the patient’s symptoms.

## Discussion

Recently, extrapulmonary TB has shown increased incidence, with or without lung involvement [8,9]. Approximately 10-11% of extrapulmonary TB cases involve joints and bones, accounting for almost 1-3% of all cases of TB, of which 50% were associated with pulmonary TB [10-12]. The overall prevalence of latent osteoarticular TB ranged from 19 to 38 million. Skeletal TB is categorized into spinal, which is reported among nearly half of cases, or arthritic with synovial disease. The other components include TB osteomyelitis and soft tissue disease due to TB [13,14]. Skeletal TB most commonly involves the spine in approximately 50% of patients, followed by the hip and knee joints.

There are many studies on osteoarticular TB, but only a few studies assessed the involvement of the foot. Ankle and foot TB (AFTB ) is a paucibacillary disease with rare positive culture. The main diagnostic method includes wound biopsy, which is confirmatory. The ultimate diagnosis of skeletal TB is based on isolation of bacteria from bone biopsy [15,16]. Clinically, noninvasive methods are recommended as diagnostic tools for AFTB, imaging studies, and laboratory findings.

Tuberculous arthritis is usually featured as being “cold,” with no edema or erythema with apparently normal skin over the infected joint. Pain is the most common complaint in cases with TB of ankle joint involvement, with minimal swelling, bone pain, restricted range of motion, limping, and muscle spasms. Systemic manifestations of TB including fever, malaise, night sweats, anorexia, and weight loss are not usually reported. With neglected cases, the patient may have complete joint degeneration and ankylosis [17,18].

The current cases had no typical clinical manifestations of TB nor any history of dyspnea, cough, night sweats, or even weight loss. It was first diagnosed as typical inflammatory arthritis and treated with anti-inflammatory drugs; therefore, minimal response was reported. The only finding that indicated the likelihood of TB-related pathology was ulcer, especially with the absence of a history of trauma and discharge. Laboratory findings, besides free chest radiography, were not specific but indicators of inflammation. MRI findings concluded the presence of joint lesion but mostly may be nonspecific. All made the initial diagnosis for TB-related arthritis difficult. Biopsy was the only diagnostic and confirmatory indication.

The literature revealed that hematological findings for musculoskeletal TB usually show decreased hemoglobin, mild lymphocytosis, and high ESR (erythrocyte sedimentation rate) [18,19]. The first detected change in plain radiography is peripheral marginal erosion of the joint. MRI of the foot reveals marginal erosion, periarticular osteoporosis, and joint space narrowing (Phemister triad). The radiological features of foot TB were difficult to distinguish from those of rheumatoid arthritis, neuropathic joints, sarcoidosis, and tumors, and therefore all should be excluded [20].

## Conclusions

Lack of awareness with rare site and ability to simulate other diseases clinically and radiologically are the main factors behind diagnostic and therapeutic delay. From a clinical point of view, it is therefore vital to highlight that tuberculous arthritis should be considered in the differential diagnosis of arthritis in a “cold” joint with biopsy in case of ulcer for confirmation. The successful treatment of skeletal TB requires early diagnosis and early anti-TB therapy. Ankle TB like other extrapulmonary variants can present nonspecific symptoms without lung involvement.
